# Quantitative Analysis of Total Aflatoxins in Dairy Cattle Feed Using a Competitive Lateral Flow Immunoassay: A Regional Study in Tigray, Ethiopia

**DOI:** 10.1002/vms3.70719

**Published:** 2025-12-02

**Authors:** Sisay Weldegebriel Zeweld, Meressa Abraha Welearegay, Enquebaher Kassaye Tarekegn

**Affiliations:** ^1^ Department of Veterinary Public Health and Food Safety Mekelle University College of Veterinary Sciences Mekelle Ethiopia; ^2^ Department of Chemistry Mekelle University College of Natural and Computational Sciences Mekelle Ethiopia

**Keywords:** aflatoxins, *Aspergillus flavus*, dairy feed, Ethiopia, feed safety, Lateral Flow Immunoassay, mycotoxin contamination, Tigray

## Abstract

Aflatoxins are toxic fungal metabolites produced mainly by *Aspergillus flavus* and *A. parasiticus*, posing serious health risks when they contaminate food and animal feed. In dairy systems, ingested aflatoxin B1 (AFB1) is metabolized in lactating cows and excreted in milk as aflatoxin M1 (AFM1), transferring the toxic burden to consumers. This study provides the first region‐specific quantitative assessment of total aflatoxin contamination in dairy cattle feed across five major towns in Tigray, northern Ethiopia: Mekelle, Wukro, Adigrat, Korem and Alamata. Feed samples were analysed using the Competitive Lateral Flow Immunoassay system. A cross‐sectional survey conducted from February to May 2025 involved 143 feed samples collected directly from farms. Substantial spatial variability in aflatoxin concentrations was observed across the study area. The mean total aflatoxin concentration was 8.84 µg/kg (SD = 15.21), with 22.4% of samples exceeding the Ethiopian regulatory limit (20 µg/kg), 29.1% surpassing the European Union threshold (5 µg/kg) and 74.1% falling below the EU limit. Roughage‐based and traditional feeds showed significantly higher contamination than commercial concentrates. Major risk factors included high storage temperature, extended storage duration, soil‐floor storage and the presence of toxigenic fungi, particularly *A. flavus* and *A. parasiticus*. Multivariate analysis confirmed fungal species type and total fungal colony count as the strongest predictors of aflatoxin levels. Alarmingly, 81.8% of farmers lacked awareness of aflatoxins, and 68.5% stored feed outdoors under unsafe conditions. Fungal isolation, species identification, toxigenicity testing and detection of aflatoxin metabolites in milk or meat were not included in this study. These findings reveal a systemic feed safety issue in the dairy sector of Tigray and indicate the need for integrated interventions, including farmer education, improved storage practices, routine monitoring and stricter regulatory enforcement. These baseline data are critical for informing local feed safety interventions and guiding aflatoxin risk management and policy development to support sustainable dairy production and public health in Ethiopia.

## Graphical abstract

Quantitative analysis of total aflatoxins in dairy cattle feed across five urban centres in Tigray, Ethiopia, revealed that 22.4% of samples exceeded national limits and 29.1% surpassed EU thresholds. Higher contamination levels were associated with roughage‐based feed, poor storage conditions and the presence of toxigenic *Aspergillus flavus* and *A. parasiticus*. Results call for integrated control measures, including farmer education, improved storage infrastructure and routine feed monitoring using rapid immunoassay tools.

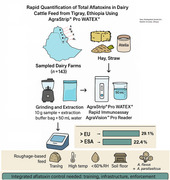

## 1 Introduction

### 1.1 Background and Justification

Aflatoxins are toxic secondary metabolites primarily produced by *Aspergillus flavus* and *A. parasiticus*, and occasionally by *A. nomius*, *A. tamarii* and *A. pseudotamarii* (Benkerroum 2020; Carvajal‐Campos et al. 2017). These fungi thrive in warm and humid environments, particularly during post‐harvest storage. Among various mycotoxins, aflatoxins are the most extensively studied due to their severe health effects, including hepatotoxicity, immunosuppression and carcinogenicity (Gurav and Medhe 2018). Structurally, aflatoxins comprise a group of closely related compounds, with aflatoxins B1, B2, G1 and G2 recognized as the most significant. The combined concentration of these four forms is referred to as total aflatoxins, a key metric in regulatory and food safety assessments. Aflatoxins commonly contaminate agricultural commodities such as cereals, oilseeds and animal feeds, with aflatoxin B1 (AFB1) being the most toxic, potent and frequently detected. Dairy cattle feeds are particularly vulnerable to contamination at various stages, including crop growth, harvesting and storage. When ingested by lactating cows, AFB1 is metabolized in the liver and excreted in milk as aflatoxin M1 (AFM1), thereby transferring the toxic burden to human consumers through the food chain (Álvarez‐Días et al. 2022; Shad et al. 2019; Feddern et al. 2013).

Several studies have reported aflatoxin concentrations in dairy feeds ranging from negligible levels to several hundred µg/kg, often exceeding established safety limits (Kortei et al. 2022; Nishimwe et al. 2020). Contamination is further exacerbated by poor post‐harvest handling practices, inadequate storage infrastructure, mechanical damage, insect activity and prolonged storage durations, all of which favour fungal growth and toxin production (Hassan et al. 2012). To mitigate these risks, many countries have established regulatory limits. The Ethiopian Standards Agency (ESA) sets the maximum allowable concentration for total aflatoxins in dairy feed at 20 µg/kg (ES 1806:2018), a level consistent with US regulations. In contrast, the European Union (EU) enforces a stricter limit of 5 µg/kg for AFB1 in feed ingredients for dairy animals (Yakubu and Vyas 2020; Mahato et al. 2019). Further confirming the contamination risk, Zeweld et al. (2025) recently isolated and characterised aflatoxigenic *Aspergillus* species from dairy feeds and storage environments across multiple towns in the Tigray region. Using traditional cultural and morphological methods, the study identified a high prevalence of *A. flavus* and other toxigenic strains, indicating that prevailing storage and environmental conditions are conducive to fungal proliferation and aflatoxin biosynthesis. These findings provide important microbiological evidence supporting the need for region‐specific aflatoxin surveillance and control measures.

Despite considerable research in eastern and central Ethiopia, there remains a significant lack of data for the northern region, particularly Tigray. For example, in Dire Dawa, feed samples averaged over 50 µg/kg, while more than 90% of milk samples from the Addis Ababa milk shed exceeded EU safety limits (FAO/WHO 2004; Geleta et al. 2024). Concentrate feeds such as oilseed cakes and agro‐industrial by‐products, frequently used in dairy rations, are especially prone to contamination when stored improperly. The economic and public health consequences of aflatoxin contamination are substantial. In livestock, it reduces productivity, increases veterinary costs and leads to market rejection of dairy products. In humans, chronic exposure to aflatoxins contributes to liver disease, impaired immune function, increased healthcare burdens and reduced labour productivity, especially in vulnerable populations (Wu 2015; Awuchi et al. 2021). Climate change, manifesting as increased temperatures, erratic rainfall and prolonged drought, further compounds aflatoxin risks in both pre‐ and post‐harvest periods (Medina et al. 2017).

Although previous studies have investigated aflatoxin contamination in other regions of Ethiopia, there are limited quantitative data specific to the Tigray region. This study provides the first region‐specific, quantitative assessment of total aflatoxin contamination in dairy cattle feed across five major towns: Mekelle, Wukro, Adigrat, Korem and Alamata. These baseline data are critical for understanding spatial variability of aflatoxin contamination in this post‐conflict area and for guiding targeted feed safety and public health interventions. Urban and peri‐urban dairy farming is rapidly expanding in these towns. However, systematic monitoring of aflatoxins in dairy feed is lacking, despite the growing use of formulated rations that incorporate high‐risk ingredients. Compounding factors such as conflict, drought, inadequate infrastructure and limited regulatory oversight further compromise feed safety, while awareness among producers and stakeholders remains low (Mengesha et al. 2024; Tesfaye et al. 2024). Recent studies from other Ethiopian regions underscore the urgency of localized surveillance. In eastern Ethiopia (Chiro, Dire Dawa and Harar), 82.8% of feed samples were contaminated, with mean concentrations of 54 µg/kg, and Dire Dawa recorded the highest levels of AFB1 (Geleta et al. 2024). In central Ethiopia (Bishoftu, Holetta and Hawassa), concentrate feeds showed higher contamination than roughages, with aflatoxin levels ranging from 1.2 to 13 µg/kg (Mengesha et al. 2024; Tadele et al. 2023). A national‐level study reported that 100% of feed and milk samples from the Greater Addis Ababa area were contaminated, with feed samples reaching concentrations as high as 549 µg/kg (Gizachew et al. 2016; Yegrem 2020). Additionally, AFB1 contamination in staple foods such as sorghum and peanuts has been reported at levels up to 738 µg/kg (Fuffa and Urga 2001).

Effective aflatoxin control requires rapid, sensitive and field‐appropriate detection tools (Sánchez‐Bayo et al. 2017). The Competitive Lateral Flow Immunoassay system (lateral flow immune‐chromatographic test [ICT]), developed by Romer Labs, utilizes a water‐based lateral flow immunoassay coupled with the AgraVision Pro Reader for fast, quantitative and user‐friendly analysis. This integrated platform enables simultaneous testing of multiple samples with minimal handling and provides results within 15 min, with detection limit as low as 2 µg/kg (Kraus et al. 2024; Al‐Jaas et al. 2023). Validated by the USDA‐FGIS and the AOAC Research Institute, the system shows strong correlation (*r* > 0.90) with conventional methods like HPLC. While it offers portability and speed, its effectiveness depends on proper sample preparation, and matrix complexity may occasionally yield false positives.

Given the expanding urban and peri‐urban dairy sector in Tigray and the increasing use of high‐risk feed ingredients, the lack of localized aflatoxin data represents a critical gap in food safety monitoring. Compounded by challenges such as conflict, drought and inadequate regulatory oversight, the region remains particularly vulnerable to aflatoxin contamination. This study addresses this gap by quantifying total aflatoxin concentrations in dairy cattle feed samples collected from five major towns in Tigray, Mekelle, Wukro, Adigrat, Korem and Alamata, using the lateral flow ICT system. The findings aim to provide essential baseline data to inform targeted risk assessments, regulatory enforcement and the development of region‐specific feed safety strategies, ultimately supporting sustainable dairy production and public health protection in Ethiopia.

### 1.2 Objective

To quantify the total aflatoxin concentrations in dairy cattle feed collected directly from farms in five major towns of the Tigray region (Mekelle, Wukro, Adigrat, Korem and Alamata) using the AgraStrip Pro WATEX rapid test system and the AgraVision Pro Reader, and to generate baseline data that can inform policymakers, feed manufacturers, veterinarians and farmers in improving feed safety, regulatory enforcement and quality control practices.

## 2 Materials and Methods

### 2.1 Study Area

The study was conducted in the urban districts of Tigray, Ethiopia, namely, Mekelle, Wukro, Adigrat, Korem and Alamata. These sites were selected based on their expanding dairy sectors and active farmer participation. The areas were classified by altitude into highland (Mekelle, Korem and Adigrat) and mid‐altitude (Alamata and Wukro) zones. According to the Tigray Bureau of Agriculture and Rural Development, there are 227 registered dairy farms in these districts: 74 in Mekelle, 32 in Wukro, 53 in Adigrat, 25 in Korem and 43 in Alamata.

### 2.2 Research Design

A cross‐sectional study was conducted from February to May 2025 to collect dairy cattle feed samples from selected farms across the five urban districts of Tigray. The primary objective was to quantify total aflatoxin concentrations in the feed samples using the lateral flow ICT system, a rapid immunoassay technique, at the Tigray Health Research Institute.

### 2.3 Sampling Technique and Sample Size Determination

A multi‐stage sampling approach was employed to select study units for the quantification of total aflatoxin concentrations in dairy cattle feed. In the first stage, five urban towns in Tigray, Mekelle, Wukro, Adigrat, Korem and Alamata, were purposively selected based on their geographic location, altitude, agro‐climatic characteristics and the presence of active and expanding dairy sectors. Background information was gathered through literature review and consultation with the Tigray Bureau of Agriculture and Rural Development. During preliminary field visits, a census of dairy farms was conducted in each town, and only farms with five or more milking cows were included in the sampling frame. The total number of eligible farms across all towns was 227. The required sample size was calculated using the Thrusfield (Thrusfield 2018) formula for an infinite population, assuming a 95% confidence level, 5% margin of error (*d*) and 50% expected prevalence (P_ex_), resulting in an initial sample size of 384.16.

$$\begin{array}{c} \mathrm{Initial}\;\mathrm{sample}\;\mathrm{size},n=\frac{Z^{2}\;x\;P_{\mathrm{ex}}\left(1-P_{\mathrm{ex}}\right)}{d^{2}}=384.16∼384\\ \mathrm{Required}\;\mathrm{sample}\;\mathrm{size},n*=\frac{1}{1/n+1/N}, \end{array}$$

where *Z* = 1.96 for 95% confidence and *N* = the total population (number of eligible farms) = 227. After adjusting for the finite population, the final sample size was determined to be 143 farms. In the second stage, proportional random sampling was used to select farms from each town based on their share of the total eligible farm population: Mekelle (47 farms), Wukro (20), Adigrat (33), Korem (16) and Alamata (27). Random selection within each town was performed using random number tables. At each selected farm, a single feed sample was collected from the existing and immediate feed type in use at the time of the visit. Feed types included hay, straw, wheat/maize middling and commercial concentrates. The samples were collected directly from storage areas or feeding troughs using sterile tools and placed in airtight, labelled containers. In addition, a structured questionnaire was administered to farm owners or attendants to collect contextual data related to feed storage practices and awareness of aflatoxin risks.

### 2.4 Laboratory Procedures

#### 2.4.1 Aflatoxin Detection Method

**FIGURE 1:**
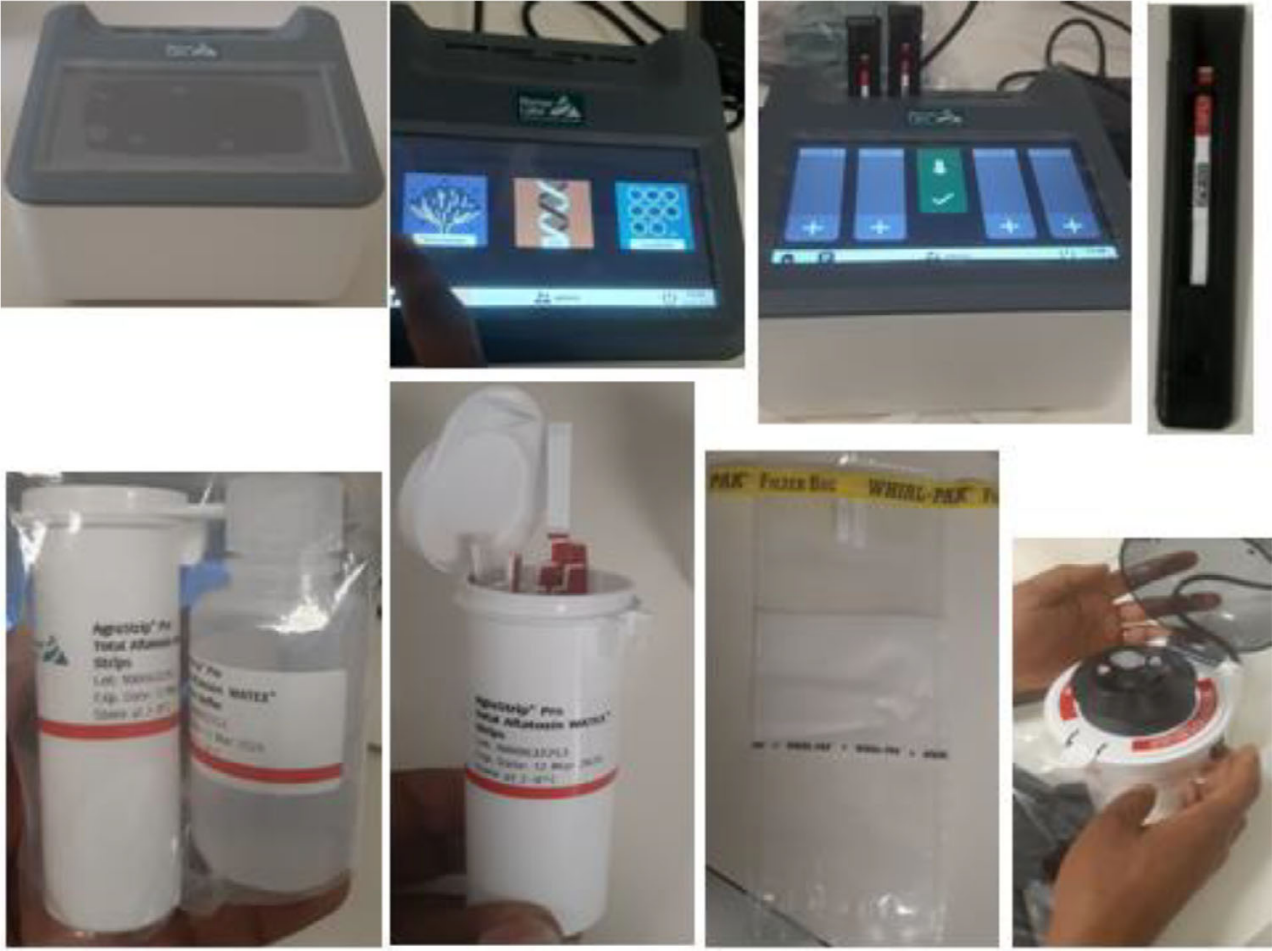
Lab setup showing the lateral flow immune‐chromatographic test for total aflatoxin quantification.

Aflatoxin quantification was performed using the rapid lateral flow immunoassay technique in combination with the AgraVision Pro Reader (Romer Labs, Austria), designed for on‐site and laboratory‐based quantitative analysis of mycotoxins in agricultural commodities (Kraus et al. 2024; Krska et al. 2012) (Figure 1).

#### 2.4.2 Sample Collection and Preparation

A total of 143 feed samples were collected directly from feed storage units or feeding areas at the farm level. Approximately 500 g of feed was obtained from each selected farm and immediately transported to the laboratory in clean, airtight containers under cool and dry conditions to prevent post‐sampling contamination and mould growth. The collected feed types included dry roughages (hay, straw and cut‐and‐carry pastures), cereal by‐products (wheat/maize middlings), commercial concentrates and *Atella* (a moist by‐product of local beer brewing). Moist samples like *Atella* were air‐dried at room temperature for 48 h before grinding to facilitate uniform particle size and ensure accurate aflatoxin extraction. Each sample was ground using a clean, electrically operated grinder suitable for dry plant materials. Grinding was performed until at least 95% of the material passed through a 20‐mesh sieve (0.84 mm), ensuring homogeneity. From the ground material, a 10 ± 0.1 g aliquot was weighed and transferred into one side of a Whirl‐Pak filter bag. One extraction buffer bag was added to the sample, followed by 50 mL of distilled water. The mixture was then sealed and shaken vigorously for 2 min at room temperature to extract aflatoxins, and allowed to settle for 1 min prior to further processing.

#### 2.4.3 Isolation, Identification and Toxigenicity of Fungal Species

Feed samples were also subjected to fungal isolation to determine the presence of aflatoxigenic species (*A. flavus* and *A. parasiticus*). The detailed procedures for fungal isolation, colony enumeration and aflatoxigenicity determination (using UV light fluorescence and ammonia vapour tests) have been fully described in our previous work (Zeweld et al. 2025). Briefly, feed samples were plated on SDA/PDA media supplemented with chloramphenicol, incubated at 27°C for 7 days and colonies were identified based on macroscopic and microscopic morphology. Aflatoxigenicity was confirmed by UV fluorescence and ammonia vapour tests.

#### 2.4.4 Extract Dilution and Centrifugation

For dilution, 1000 µL of the provided dilution buffer was pipetted into a microcentrifuge tube, and 100 µL of the feed extract was added and mixed thoroughly. The mixture was centrifuged for 30 s to separate particulate matter. The supernatant was carefully collected for further testing.

#### 2.4.5 Aflatoxin Quantification Using AgraVision Pro Reader

The AgraVision Pro Reader was turned on and calibrated using the QR code card specific to the test kit lot. Test strips were placed into the designated cartridges and inserted into the reader. Sample identification (ID, matrix type and quantitation range) was entered into the system. Upon prompt by the reader (indicated by a flashing drop symbol), 100 µL of the centrifuged sample supernatant was added to the cartridge. The reader automatically initiated the assay, and results were displayed after incubation. The test is based on a competitive immunoassay principle, where aflatoxin present in the sample competes with the test line‐bound antigen for binding to gold‐conjugated monoclonal antibodies. The signal intensity is inversely proportional to the aflatoxin concentration, which is quantitatively interpreted by the reader.

#### 2.4.6 Questionnaire Survey

A structured questionnaire was developed specifically for this study to collect data on factors potentially influencing the growth of aflatoxigenic fungi and the quantification of total aflatoxin levels in dairy cattle feed. The English‐language version of the questionnaire is provided as a Supporting Information. The survey included categorical independent variables such as farm location, farm owner's gender, education level, dairy management training, farm altitude, knowledge of mould and aflatoxins, feed type, grazing system, feed storage duration and storage conditions. These variables were investigated for their potential association with total aflatoxin concentrations measured using the lateral flow ICT kit. Continuous covariates, including the average ambient temperature of the study area, feed storage area temperature (°C) and feed moisture content (%), were also recorded, as these environmental and management‐related factors can significantly influence fungal proliferation and aflatoxin biosynthesis. The dependent variable was the quantified total aflatoxin level in µg/kg, serving as the main outcome of interest in relation to the surveyed risk factors.

### 2.5 Quality Control and Storage

All analyses were performed in strict accordance with the manufacturer's instructions as outlined in the Romer Labs product leaflet (https://www.romerlabs.com) to ensure the accuracy, reliability and reproducibility of results. Test kits and dilution buffers were stored at 6°C, while extraction buffer bags and other consumables were maintained at room temperature (approximately 25°C). To avoid cross‐contamination and ensure consistency, components from different lot numbers were not mixed. All disposable materials used during the analysis were handled following standard biosafety procedures and disposed of as biohazard waste.

### 2.6 Statistical Analysis

Data analysis was carried out using SPSS version 20 (IBM Corp., Armonk, NY, USA). Descriptive statistics were employed to summarise the total aflatoxin concentration levels across different towns and feed types. Categorical variables such as storage method, feed type and contamination status were presented using frequencies and percentage distributions. Prior to inferential analysis, assumptions of parametric tests, including normality, homogeneity of variance and linearity, were assessed using appropriate diagnostic tools. These assumptions were met, supporting the use of parametric methods. One‐way analysis of variance (ANOVA) was used to determine statistically significant differences in aflatoxin levels among towns, feed types and other categorical factors. Eta squared (*η* ^2^) was calculated to estimate effect sizes. Chi‐square tests were performed to examine associations between categorical predictors (e.g., storage method) and contamination status, defined based on whether samples exceeded the ESA regulatory threshold of 20 µg/kg. Additionally, multivariate analyses were conducted to control for potential confounders. Analysis of covariance (ANCOVA) was applied to evaluate the effect of fixed factors while adjusting for covariates. Multiple linear regression was used to assess the influence of continuous variables such as storage temperature, feed moisture content and fungal colony count on aflatoxin levels. All statistical tests were two‐tailed, and a *p*‐value of less than 0.05 was considered statistically significant.

## 3 Results

### 3.1 Descriptive Analysis of Total Aflatoxin Levels in Dairy Feed Across Categorical Variables

The overall mean total aflatoxin level detected across all dairy feed samples (*N* = 143) was 8.84 µg/kg (SD = 15.21), indicating high variability in aflatoxin contamination among different sample sources and conditions. Aflatoxin contamination levels varied considerably across the five study areas. The highest mean aflatoxin level was observed in Korem Town (16.956 µg/kg, SD = 17.78), followed by Alamata Town (11.33 µg/kg, SD = 16.9762) and Adigrat Town (9.01 µg/kg, SD = 16.24). Lower contamination levels were recorded in Mekelle City (6.92 µg/kg, SD = 14.02) and Wukro Town (3.21 µg/kg, SD = 7.68). These differences may reflect varying environmental conditions, feed management practices or storage infrastructure. When stratified by geographical zones, dairy feed samples from the highland zone had slightly higher aflatoxin levels (9.31 µg/kg, SD = 15.71) than those from middle altitude areas (7.87 µg/kg, SD = 14.27), although this difference was not statistically significant in the ANOVA (*p* = 0.60) (Table 1).

**TABLE 1:** Descriptive statistics of total aflatoxin levels (µg/kg) in dairy feed by different categorical variables of interest (*N* = 143).

Variables	*N*	Mean (µg/kg)		Std. deviation
Study area				
Mekelle City	47	6.92		14.02
Wukro Town	20	3.21		7.68
Adigrat Town	33	9.01		16.24
Korem Town	16	16.96		17.78
Alamata Town	27	11.33		16.98
Geographical zone				
Highland	96	9.31		15.71
Middle altitude	47	7.87		14.27
Type of feed provided				
Roughage, grain by‐products, cut and carry pasture and *Atella*	116		10.48	16.44
Commercial concentrates	27		1.76	2.19
RH% condition of storage compartment				
Ideal RH (50%–60%)	38		0.00	0.00
High RH (> 60%)	105		12.034	16.65
Sample type				
Commercial concentrates	23		2.80	8.45
Hay	49		12.20	16.92
Straw	33		10.76	17.41
Wheat/maize middlings	38		6.48	12.87
Type of floor				
Soil floor	94		12.70	17.32
Concrete floor	49		1.43	4.33
Length of feed storage period				
Less than 3 months	25		0.00	0.00
3–6 months	36		8.96	15.98
Greater than 6 months	82		11.48	16.26
Nature of detected fungal species				
Aflatoxigenic species	48		26.33	15.14
Non‐aflatoxigenic *A. flavus*, *A. parasiticus* and others	95		0.000	0.00
Type of fungal species detected				
*A. flavus* only	13		18.92	10.81
*A. parasiticus* only	7		19.70	10.71
Both *A. flavus* and *A. parasiticus*	28		31.42	16.02
Non‐aflatoxigenic *A. flavus* and *A. parasiticus*	24		0.00	0.00
Other fungal species	71		0.00	0.00
Total	143		8.84	15.21

Feeds composed of roughage, grain by‐products, cut‐and‐carry pasture and Atella were associated with substantially higher aflatoxin levels (10.48 µg/kg, SD = 16.44) compared to commercial concentrates (1.76 µg/kg, SD = 2.19). Feed stored under high relative humidity (RH) conditions (> 60%) showed significantly elevated aflatoxin concentrations (12.03 µg/kg, SD = 16.65), whereas samples stored under ideal RH (50%–60%) exhibited no detectable aflatoxin contamination (0.00 µg/kg, SD = 0.00). Among feed types, hay exhibited the highest mean aflatoxin level (12.20 µg/kg, SD = 16.92), followed by straw (10.76 µg/kg, SD = 17.41), wheat/maize middlings (6.48 µg/kg, SD = 12.87) and commercial concentrates (2.80 µg/kg, SD = 8.45) (Table 1).

**FIGURE 2:**
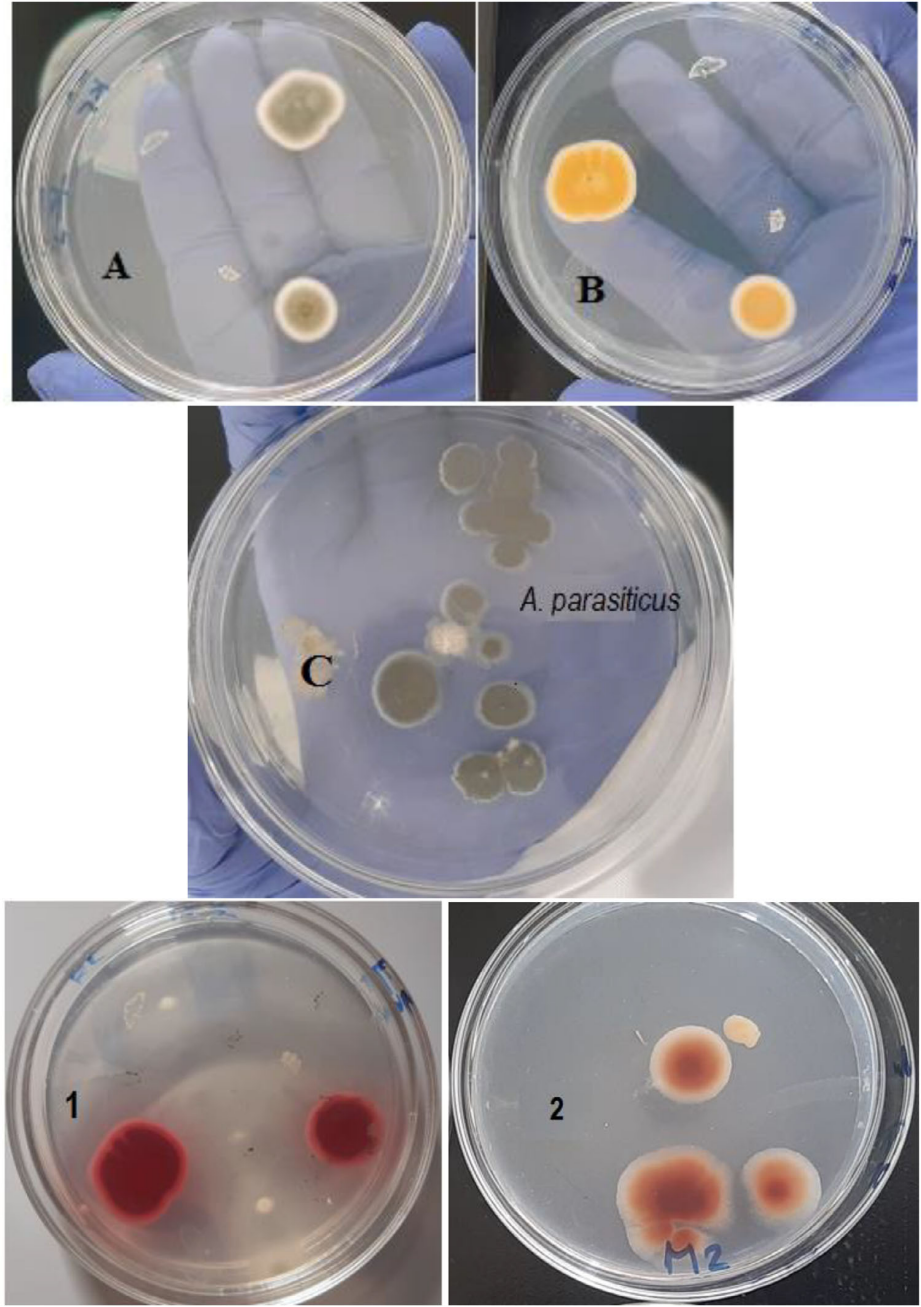
Macroscopic morphological characteristics and ammonia vapour test results of *Aspergillus* isolates. (A) Surface colony of *A. flavus* showing yellow‐green pigmentation, (B) reverse colony showing pale‐brown to golden colouration on PDA plate and (C) *A. parasiticus* colony showing a dark green to olive‐green surface with a white to pale margin, (1) ammonia vapour test of *A. flavus*, and (2) ammonia vapour test of *A. parasiticus* after 30 min of exposure.

**FIGURE 3:**
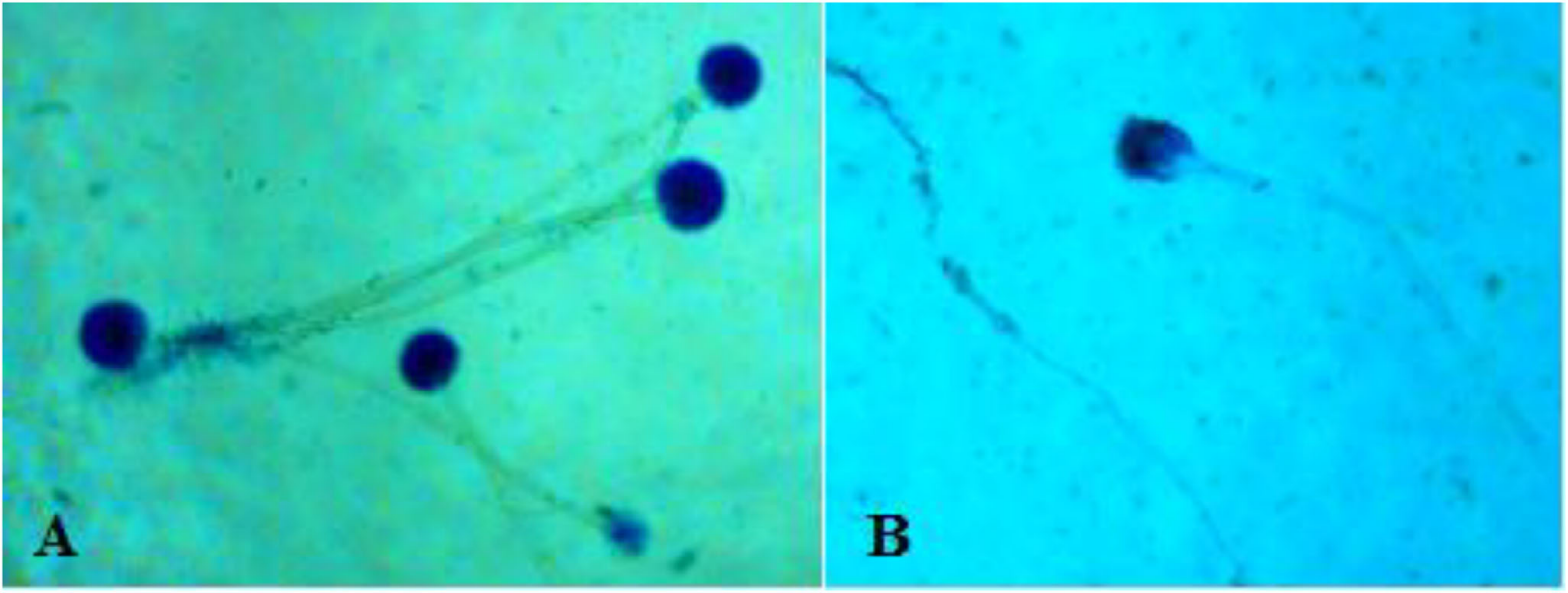
Microscopic appearances of *A. flavus* (A) with globose or spherical conidial heads and *A. parasiticus* (B) with oval conidial heads, showing conidiophore and conidial head structures, stained with lactophenol cotton blue (100×).

The type of floor in storage facilities also had a marked influence. Samples stored on soil floors contained significantly higher aflatoxin levels (12.70 µg/kg, SD = 17.32) compared to those stored on concrete floors (1.43 µg/kg, SD = 4.33). No aflatoxin was detected in feeds stored for less than 3 months (0.00 µg/kg, SD = 0.00). In contrast, feeds stored for 3–6 months had mean aflatoxin levels of 8.96 µg/kg (SD = 15.98), and those stored for over 6 months reached 11.48 µg/kg (SD = 16.26). Samples contaminated with aflatoxigenic fungal species had a mean aflatoxin level of 26.33 µg/kg (SD = 15.14), whereas those associated with non‐aflatoxigenic fungi contained no detectable aflatoxin (0.00 µg/kg, SD = 0.00). Further, among the types of fungal species, samples where both *A. flavus* and *A. parasiticus* (Figures 2 and 3) were detected had the highest mean aflatoxin concentration (31.42 µg/kg, SD = 16.02), followed by those with either *A. parasiticus* only (19.70 µg/kg, SD = 10.71) or *A. flavus* only (18.92 µg/kg, SD = 10.81). In contrast, samples with non‐aflatoxigenic *Aspergillus* spp. or other fungal species showed no aflatoxin presence (0.00 µg/kg, SD = 0.00) (Table 1).

For some parameters in Tables 1 and 2, the standard deviations exceeded the mean values (e.g., Table 2: mean = 0.45 µg/kg, SD = 1.33 µg/kg). This high variability reflects the heterogeneous nature of the feed samples, where a few samples had extremely high aflatoxin levels while many others were near or below the detection limits, resulting in a positively skewed distribution. Such differences likely arise from variations in feed storage, handling practices and environmental conditions across the study sites.

**TABLE 2:** Mean total aflatoxin levels in dairy feeds by regulatory limit categories.

Regulatory limit category	*N*	Mean (µg/kg)	Std. deviation	Percent
Below EU limit (< 5 µg/kg)	106	0.45	1.33	74.1%
Between EU and ESA limits (5–20 µg/kg)	5	14.04	1.84	3.5%
Above ESA limit (> 20 µg/kg)	32	35.81	7.59	22.4%
**Total**	**143**	**8.84**	**15.21**	**100%**

### 3.2 Proportion of Samples Exceeding Regulatory Limits

**FIGURE 4:**
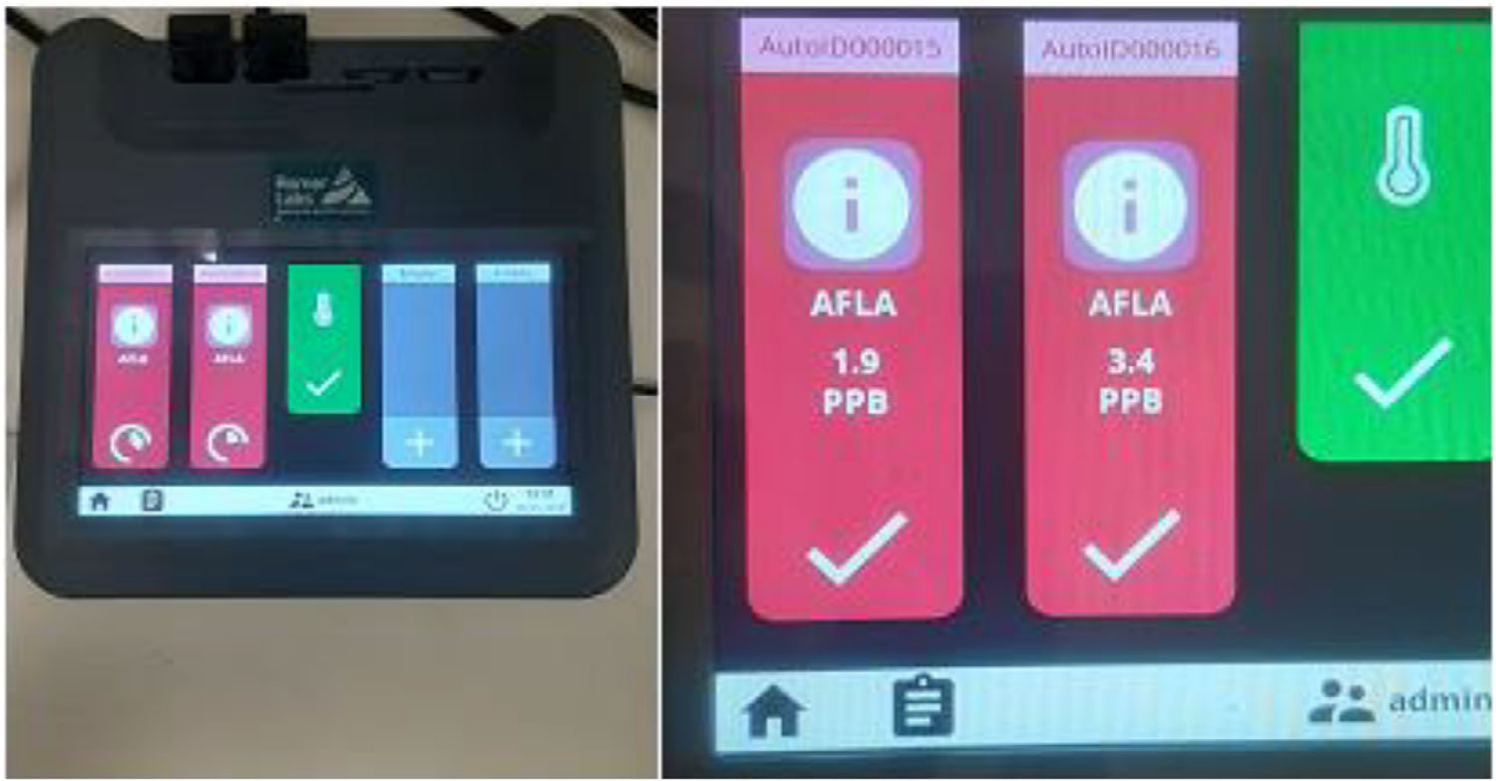
AgraVision Pro Reader displaying a reading below the EU aflatoxin limit. NB: 1 ppb = 1 µg/kg.

A one‐way ANOVA was conducted to compare the mean total aflatoxin levels in dairy feed samples categorized by regulatory limits: below the EU threshold (< 5 µg/kg; 74.1% of the samples), between the EU and ESA thresholds (5–20 µg/kg; 3.5% of the samples) and above the ESA limit (> 20 µg/kg; 22.4% of the samples). The analysis included 143 samples, and the results revealed a statistically significant difference in aflatoxin concentrations among the three groups (*F*(2, 140) = 1088.71, *p* < 0.001). The mean aflatoxin levels were 0.45 µg/kg for samples below the EU limit (Figure 4), 14.04 µg/kg for those between 5 and 20 µg/kg and 35.81 µg/kg for samples exceeding 20 µg/kg. The overall mean was 8.84 µg/kg with a standard deviation of 15.21 (Table 2).

### 3.3 Determinants of Aflatoxin Levels in Dairy Feed

The one‐way ANOVA analysis revealed significant differences in total aflatoxin levels across various categorical variables of interest, with effect sizes quantified using eta squared (*η* ^2^) to interpret the proportion of variance explained by each factor. The study area showed a marginally non‐significant difference in total aflatoxin levels among the five locations assessed (*F*(4, 138) = 2.3, *p* = 0.064). Although the *p*‐value was slightly above the conventional threshold for statistical significance, the effect size (*η* ^2^ = 0.062) suggests a small to moderate proportion of the variance in aflatoxin levels (6.2%) was attributable to the study area. When data were aggregated by geographical zone (highland vs. lowland), no significant differences were observed in aflatoxin levels (*F*(1, 141) = 0.3, *p* = 0.598), and the corresponding effect size was negligible (*η* ^2^ = 0.002), indicating only 0.2% of the variance was explained by geographical zone (Table 3).

**TABLE 3:** One‐way ANOVA summary for total aflatoxin level by different categorical variables of interest.

Source of variation	Sum of squares	df	Mean square	*F*	*p*‐value	Eta		Eta squared (*η* ^2^)
Study area								
Between groups	2031.52	4	507.88	2.3	0.064	0.249		0.062
Within groups	30,836.40	138	223.45					
Geographical zone								
Between groups	65.06	1	65.06	0.3	0.598	0.044		0.002
Within groups	32,802.85	141	232.64					
Type of feed								
Between groups	1668.81	1	1668.81	7.5	0.007	0.225	0.051	
Within groups	31,199.10	141	221.27					
Feed storage RH%								
Between groups	4040.89	1	4040.89	19.8	0.000	0.351	0.123	
Within groups	28,827.02	141	204.45					
Sample type								
Between groups	1723.48	3	574.49	2.6	0.057	0.229	0.052	
Within groups	31,144.44	139	224.06					
Type of floor								
Between groups	4086.05	1	4086.05	20.0	< 0.001	0.353	0.124	
Within groups	28,781.87	141	204.13					
Length of feed storage period								
Between groups	2524.28	2	1262.14	5.8	0.004	0.277	0.077	
Within groups	30,343.63	140	216.74					
Nature of the detected fungal species								
Between groups	22,098.64	1	22,098.64	289.3	0.000	0.820	0.672	
Within groups	10,769.27	141	76.38					
Type of fungal species detected								
Between groups	23,844.37	4	5961.10	91.2	0.000	0.852	0.725	
Within groups	9023.54	138	65.39					
**Total**	**32,867.911**	**142**						

A significant difference in aflatoxin levels was detected across different types of feed (*F*(1, 141) = 7.5, *p* = 0.007), with an effect size of *η* ^2^ = 0.051, signifying that feed type accounted for 5.1% of the observed variance. This suggests that certain feed compositions may predispose to higher aflatoxin contamination. The RH% of feed storage exhibited a highly significant influence on aflatoxin levels (*F*(1, 141) = 19.8, *p* < 0.001). The effect size (*η* ^2^ = 0.123) indicated that feed storage humidity accounted for 12.3% of the variance, underscoring its substantial role in aflatoxin proliferation. Analysis based on sample type (e.g., compound feed, grain or ingredients) showed a borderline significant difference (*F*(3, 139) = 2.6, *p* = 0.057), with a moderate effect size (*η* ^2^ = 0.052), suggesting that sample type may modestly influence aflatoxin concentration. There was a statistically significant difference in aflatoxin levels based on type of floor used in storage facilities (*F*(1, 141) = 20.0, *p* < 0.001), with an effect size of *η* ^2^ = 0.124. This indicates that flooring type explained 12.4% of the variance, reflecting its critical role in maintaining hygienic feed storage conditions. The length of feed storage period also showed a significant effect (*F*(2, 140) = 5.8, *p* = 0.004), accounting for 7.7% of the variance (*η* ^2^ = 0.077). This finding highlights the risk associated with prolonged storage, likely due to increased opportunity for fungal growth and aflatoxin accumulation (Table 3).

Remarkably, the nature of the detected fungal species (e.g., toxigenic vs. non‐toxigenic) had a profound impact on aflatoxin levels (*F*(1, 141) = 289.3, *p* < 0.001). The effect size was extremely large (*η* ^2^ = 0.672), indicating that 67.2% of the variance in aflatoxin levels could be attributed to fungal toxigenicity. Similarly, the type of fungal species detected yielded a highly significant effect (*F*(4, 138) = 91.2, *p* < 0.001), with an even greater effect size (*η* ^2^ = 0.725). This suggests that the specific fungal species present explained 72.5% of the variation in aflatoxin concentration, affirming the central role of species‐specific mycotoxin production (Table 3).

### 3.4 ANCOVA and Multiple Regression of Factors Influencing Total Aflatoxin Levels

The ANCOVA revealed a statistically significant overall model predicting total aflatoxin levels based on several categorical factors (*F*(16, 126) = 40.532, *p* < 0.001, partial *η* ^2^ = 0.837), indicating that 83.7% of the total variability in the dependent variable was accounted for by the covariate and fixed factors in the model. After adjusting for covariates, the location of sample collection remained a significant predictor of aflatoxin levels (*F*(3, 126) = 2.689, *p* = 0.049, partial *η* ^2^ = 0.060). Post hoc pairwise comparisons revealed that samples from Adigrat had significantly higher aflatoxin levels than those from Mekelle (mean difference = −6.872 µg/kg, *p* = 0.021) and Korem (mean difference = −6.180 µg/kg, *p* = 0.058), while samples from Alamata had significantly lower levels compared to Wukro (mean difference = −7.032 µg/kg, *p* = 0.010). These findings underscore the presence of localized differences in contamination, likely linked to variations in feed management and storage conditions. The type of feed routinely provided continued to show a strong and statistically significant effect (*F*(1, 126) = 48.774, *p* < 0.001, partial *η* ^2^ = 0.279) (Table 4).

**TABLE 4:** ANCOVA summary table for categorical variables influencing total aflatoxin levels (µg/kg).

Source	Sum of squares	df	Mean square	*F*	*p*‐value	Partial Eta squared
Corrected model	10,673.22	16	667.08	41.514	< 0.001	0.837
Intercept	4317.21	1	4317.21	268.499	< 0.001	0.676
Study area	129.34	3	43.12	2.689	0.049	0.060
Type of feed provided	786.36	1	786.36	48.774	< 0.001	0.279
RH% of feed storage compartment	7.37	1	7.37	0.457	0.500	0.004
Sample type	1.11	3	0.37	0.091	0.965	0.002
Type of storage area	1.09	1	1.09	0.067	0.797	0.001
Feed storage period	118.20	2	59.10	3.658	0.029	0.055
Type of species	1970.91	3	656.97	12.218	< 0.001	0.225
Error	2074.61	129	16.08	—	—	—
Total	35,248.00	146	—	—	—	—
Corrected Total	12747.83	145	—	—	—	—
R^2^	0.837	—	—	—	—	—
Adjusted R^2^	0.817	—	—	—	—	—
Levene's test of equality of error variances (F(88, 54))	—	—	—	—	6.951	0.001

*Note*: *R* ^2^ = 0.837, adjusted *R* ^2^ = 0.817, Levene's test of equality of error variances: *F*(88, 54) = 6.951, *p* = 0.000.

In contrast to the univariate findings, the RH% of the feed storage area no longer had a statistically significant effect (*F*(1, 126) = 0.457, *p* = 0.500, partial *η* ^2^ = 0.004). Although high RH (> 60%) is generally considered conducive for fungal growth, its independent contribution to aflatoxin levels appears to diminish when controlling for other variables such as fungal load and feed type. Similarly, after adjusting for covariates, Sample Type did not exhibit a statistically significant influence on aflatoxin levels (*F*(3, 129) = 0.091, *p* = 0.965, partial *η* ^2^ = 0.002). Likewise, Type of Storage Area showed no significant effect (*F*(1, 129) = 0.067, *p* = 0.797, partial *η* ^2^ = 0.001), suggesting these factors may not play a direct role when analysed alongside stronger predictors. However, the feed storage period retained its statistical significance (*F*(2, 129) = 3.658, *p* = 0.029, partial *η* ^2^ = 0.055), indicating that longer storage durations remain a risk factor for aflatoxin accumulation even after adjusting for other variables. Additionally, the type of species for which the feed was intended had a highly significant effect (*F*(3, 129) = 12.218, *p* < 0.001, partial *η* ^2^ = 0.225), possibly due to differences in feed formulation and storage practices among species. A multiple linear regression analysis was also conducted to evaluate the effects of continuous variables. The overall regression model was statistically significant (*F*(3, 139) = 16.390, *p* < 0.001), explaining approximately 26.1% of the variance in total aflatoxin levels (*R* ^2^ = 0.261, Adjusted *R* ^2^ = 0.245). Among the predictors, total fungal colony count emerged as the strongest contributor (*β* = 0.665, *t* = 6.257, *p* < 0.001), followed by storage temperature (*β* = 0.260, *t* = 2.495, *p* = 0.014). Moisture content, however, was not a significant predictor (*β* = −0.005, *t* = −0.065, *p* = 0.948), indicating a limited role when fungal load and temperature are simultaneously considered (Table 4).

A multiple linear regression analysis was conducted to examine the effect of temperature of the storage environment, moisture content of the dairy feed and total fungal colony count (continuous covariates) on total aflatoxin levels (continuous dependent variable). The model was statistically significant (*F*(3, 139) = 16.390, *p* < 0.001) and explained approximately 26.1% of the variance in aflatoxin contamination (*R* ^2^ = 0.261, Adjusted *R* ^2^ = 0.245, standard error of the estimate = 13.22). Among the predictors, total fungal colony count was the strongest and most significant contributor to aflatoxin levels (*β* = 0.665, *t* = 6.257, *p* < 0.001), suggesting that fungal load plays a central role in aflatoxin biosynthesis. Additionally, temperature of the feed storage area had a significant positive effect (*β* = 0.260, *t* = 2.495, *p* = 0.014), indicating that higher storage temperatures are associated with increased aflatoxin levels. However, the moisture content of the dairy feed (ranged from 50% to 75%) was not a significant predictor (*β* = −0.005, *t* = −0.065, *p* = 0.948), suggesting that its influence may be minimal or context‐dependent when fungal load and temperature are also considered in the model (Table 5).

**TABLE 5:** Multiple linear regression analysis predicting total aflatoxin levels (µg/kg).

Predictor variable	*β* (unstandardized coefficient)	SE	*β* (standardized coefficient)	*t*	*p*‐value
Constant (intercept)	−104.69	24.52	—	−4.270	< 0.001
Temperature of the dairy feed storage environment (°C)	1.41	0.57	0.26	2.495	0.014
Moisture content of the dairy feed (50%–75%)	−0.03	0.42	−0.01	−0.065	0.948
Total fungal colony count (CFU/g)	0.00	0.00	0.67	6.257	< 0.001
R^2^	0.261	—	—	—	—
Adjusted R	0.245	—	—	—	—
Levene's test of equality of error variances (F(3, 139))	—	—	—	16.39	<0.001

*Note*: *R* = 0.511, *R* ^2^ = 0.261, adjusted *R* ^2^ = 0.245, *F*(3, 139) = 16.39, *p* < 0.001.

The overall regression model was statistically significant, *F*(3, 139) = 16.39, *p* < 0.001, explaining approximately 26.1% of the variance in total aflatoxin levels (*R* ^2^ = 0.261). Among the predictors, both storage temperature and fungal colony count significantly predicted aflatoxin levels, while moisture content did not.

### 3.5 Result of Questionnaire Survey

The survey conducted across five towns indicated a pronounced gender disparity among dairy farm owners, with males comprising the majority (88.1%). Educational attainment was generally low, and only a minority had received formal training in dairy management (27.3%). Most farms relied on locally available feed resources and adopted a zero‐grazing system (69.9%). However, suboptimal feed storage practices were prevalent, with 65.7% using soil floors and 68.5% storing feed outdoors, heightening the risk of contamination. Awareness of mould and aflatoxin hazards was limited (18.2%), and 57.3% of respondents stored feed for over 6 months. These findings highlight critical gaps in training, gender inclusivity and feed safety, potentially undermining milk quality and dairy productivity (Table 6).

**TABLE 6:** Demographic and farm management characteristics of dairy farm owners.

Variable	Category	Frequency	Percent
Gender of farm owner	Male	126	88.1
	Female	17	11.9
Study areas	Mekelle city	47	32.9
	Wukro town	20	14.0
	Adigrat town	33	23.1
	Korem town	16	11.2
	Alamata town	27	18.9
Educational status of dairy farm owners	Graduate	20	14.0
	Secondary school	29	20.3
	Primary school	58	40.6
	Illiterate	36	25.2
Previous training on dairy farm management	Yes	39	27.3
	No	104	72.7
The type of feed routinely provided to dairy cows	Roughage, grain by‐products, cut and carry pasture, Atella	116	81.1
	Commercial concentrates	27	18.9
Type of floor of storage area	Soil floor	94	65.7
	Concrete floor	49	34.3
Knowledge of dairy farm owners about mould or aflatoxin	Yes	26	18.2
	No	117	81.8
Nature of feed storage environment	Indoor storage	45	31.5
	Outdoor storage	98	68.5
Type of grazing system	Zero‐grazing	100	69.9
	Open grazing	13	9.1
	Semi‐intensive grazing	30	21.0
Length of feed storage period	Less than 3 months	25	17.5
	3–6 months	36	25.2
	Greater than 6 months	82	57.3

## 4 Discussion

This study extends our previous study (Zeweld et al. 2025) on the detection of aflatoxigenic fungi in dairy feeds by quantifying the aflatoxin burden. While the earlier study established the prevalence and risk factors for fungal contamination, the present study demonstrates the extent of toxin production, thereby providing a more comprehensive risk assessment for dairy production and public health.

This study provides the first region‐specific, quantitative assessment of total aflatoxin contamination in dairy cattle feed across five major towns, Mekelle, Wukro, Adigrat, Alamata and Korem, in the Tigray region of northern Ethiopia. Using the lateral flow ICT system, we generated critical baseline data revealing substantial spatial variability in aflatoxin concentrations and clear associations with environmental conditions, fungal species composition, feed type and storage practices. Although the overall mean aflatoxin level (8.84 µg/kg) remained below the national regulatory threshold (20 µg/kg), 22.4% of samples exceeded this limit, while 29.1% surpassed the more stringent European Union standard of 5 µg/kg. These findings demonstrate that aflatoxin risk is not limited to sporadic incidents but is instead a systemic issue embedded in the structural, biological and managerial dimensions of the dairy value chain of the region. The pronounced heterogeneity across feed types and handling conditions indicates both the complexity of aflatoxin contamination and the urgency of implementing targeted control strategies. In the following discussion, we interpret these findings in the context of existing literature, identify potential sources of contamination and explore their broader implications for feed safety, dairy productivity and public health.

Environmental and infrastructural factors strongly influenced aflatoxin levels. Dairy feed stored under high RH (> 60%) and on soil floors exhibited significantly higher contamination levels (12.034 and 12.696 µg/kg, respectively), indicating poor storage conditions as critical risk factors. Similar patterns have been reported in southern Ethiopia (Bereka et al. 2022), where storage under humid and unsealed conditions promoted fungal proliferation and toxin accumulation. Likewise, Motbaynor Wubaye et al. (2021) identified soil contact and inadequate ventilation as major drivers of AFB1 contamination in cereal grains and feed. Prolonged storage emerged as another key determinant. In our study, no aflatoxin was detected in feeds stored for less than 3 months, while samples stored over 6 months had a mean concentration of 11.477 µg/kg. This aligns with the findings of Chala et al. (2013) and Mohammed et al. (2016), who documented a strong time‐dependent increase in aflatoxin levels under typical Ethiopian postharvest conditions. Together, these findings show the importance of limiting storage duration, improving ventilation and avoiding soil‐based or outdoor storage to mitigate aflatoxin risks.

The most significant predictor of aflatoxin contamination was the presence of aflatoxigenic fungal species. Feed samples containing both *A. flavus* and *A. parasiticus* showed extremely high contamination levels (mean = 31.418 µg/kg), far exceeding both EU and Ethiopian thresholds. In contrast, feeds colonised by non‐toxigenic fungi exhibited no detectable aflatoxins. These findings echo previous Ethiopian studies (Zeweld et al. 2025; Assaye et al. 2016) and support the biological specificity of aflatoxin production. Multivariate analysis confirmed that fungal species type accounted for the largest proportion of explained variance (*η* ^2^ = 0.725), far exceeding that of environmental variables or feed composition. Similar results have been reported by Mohammed et al. (2021), emphasising the critical need for species‐level monitoring in aflatoxin surveillance programmmes.

Feed type was another significant determinant. Traditional feedstuffs composed of roughage, grain by‐products, cut‐and‐carry pasture and *Atella* were associated with significantly higher aflatoxin levels than commercial concentrates (mean difference = 12.204 µg/kg, *p* < 0.001). These findings are consistent with those of Mengesha et al. (2024) and Fikadu et al. (2022), who found that local feed mixtures in Addis Ababa and Oromia were more susceptible to contamination due to poor handling, inadequate drying and lack of formal quality control. The higher vulnerability of traditional feed is likely due to the diversity of ingredients, extended storage periods and lack of uniformity in moisture content and packaging. Although not always statistically significant in univariate models, ANCOVA revealed that geographic differences or sample location remained a significant predictor after controlling for covariates (*F*(3, 126) = 2.689, *p* = 0.049). Specifically, Adigrat samples were significantly more contaminated than those from Mekelle and Korem. This likely reflects localised differences in storage infrastructure, climatic conditions and fungal ecology, similar to disparities reported by Tefera et al. (2022) and Gizachew et al. (2016) in other parts of Ethiopia.

Total fungal colony count was the strongest predictor of aflatoxin concentration (*β* = 0.665, *p* < 0.001), followed by storage temperature (*β* = 0.260, *p* = 0.014). These results align with those of Medina et al. (2017) and Hassan et al. (2012), who showed that fungal proliferation and aflatoxin biosynthesis are strongly enhanced at higher temperatures and under sufficient substrate availability. Although moisture is a well‐established factor influencing mould growth and aflatoxin production, our study did not find a significant association between feed moisture content and aflatoxin levels. This may be attributable to the relatively narrow range of moisture values across the sampled feeds, which were largely within levels conducive to fungal growth. Furthermore, aflatoxin accumulation is a multifactorial process influenced by interactions among temperature, fungal species, feed composition and storage practices. Therefore, the effect of moisture alone may be less apparent in field conditions, indicating the complexity of factors governing aflatoxin contamination. Tefera et al. (2022) and Ayelign and De Saeger (2020) similarly found that while moisture enables fungal growth, its individual predictive value diminishes when fungal burden is accounted for. These findings advocate for risk assessment frameworks that prioritise biological over purely environmental indicators.

While the mean total aflatoxin concentration detected in this study (8.84 µg/kg) was below the national limit of 20 µg/kg (ES 1806:2018) of Ethiopia, 22.4% of samples exceeded this threshold and 29.1% surpassed the EU maximum allowable level of 5 µg/kg. This level of non‐compliance aligns with findings from Addis Ababa and Bishoftu, where 29% and 33% of dairy feed samples, respectively, exceeded the national limit (Mengesha et al. 2024; Tadele et al. 2023).

A study conducted in Kenya reported that 70% of animal feed samples had aflatoxin levels below the EU limit of 5 µg/kg, indicating a comparable level of contamination control across different regions (Kemboi et al. 2020). The pattern observed in the study of urban districts of Tigray is consistent with national findings. Mengesha et al. (2024) reported that 29% of dairy feed samples in Addis Ababa exceeded 20 µg/kg, while Tefera et al. (2022) documented exceedance rates as high as 40% in Sidama Zone during the wet season. Similarly, Gizachew et al. (2016) found AFB1 levels reaching up to 549 µg/kg in dairy feed from the central highlands, far above regulatory limits. These data collectively suggest that aflatoxin contamination is not an isolated phenomenon but a systemic issue affecting various regions and production systems across Ethiopia.

The multiple linear regression model explained 26.1% of the variance in total aflatoxin levels, indicating that the included variables accounted for a portion of the observed contamination. While this proportion may seem modest, aflatoxin contamination in dairy feed is influenced by a wide range of factors beyond the measured variables, including microclimatic conditions, fungal strain variability, interactions between different feed ingredients and handling practices at farm and market levels. Therefore, it is expected that a substantial portion of the variability (approximately 73.9%) remains unexplained by the current model. Similar findings (Tefera et al. 2022; Ayelign and De Saeger 2020) have been reported in other studies, where aflatoxin levels exhibited high heterogeneity due to complex environmental and management factors. This indicates the need for future studies to include additional predictors, such as feed moisture at storage, temperature fluctuations and fungal load, to improve the predictive capacity of aflatoxin contamination models.

In addition to biological and environmental factors, socio‐technical limitations significantly contribute to contamination risk. The present survey data showed that 81.8% of farmers lacked awareness of aflatoxins, 65.7% stored feed on soil floors and 68.5% used outdoor storage. Furthermore, 57.3% stored feed for over 6 months, and only 27.3% had received any formal training. These findings mirror those of Guchi (2015) and Toma (2019), who noted widespread gaps in aflatoxin knowledge and practice among Ethiopian smallholders.

Inadequate infrastructure and lack of extension support were also noted in studies from southern Ethiopia (Bereka et al. 2022) and central Nigeria (Ezekiel et al. 2014), suggesting a regional pattern. Such socio‐technical vulnerabilities undermine the effectiveness of even well‐designed biological and chemical interventions, emphasising the need for integrated capacity‐building, access to safe storage materials and community‐based risk communication. This study demonstrates that aflatoxin contamination in dairy feed is a multifactorial issue driven by synergistic effects of fungal ecology, environmental stressors, feed composition and socio‐institutional deficits. While fungal species and feed type were the dominant predictors of contamination, environmental parameters such as temperature, humidity and storage structure also contributed substantially. However, the persistence of unsafe practices and low awareness among farmers remains a critical barrier to progress. Addressing aflatoxin risks in Ethiopia requires a systems‐level approach that combines regulatory enforcement, fungal monitoring, infrastructure improvement and farmer training. Emphasis should be placed on fungal species surveillance, safe feed storage protocols and education campaigns tailored to the local context. Without concurrent investment in both technical and human capacity, aflatoxin contamination will continue to threaten food safety, public health and the dairy economy.

## 5 Conclusion and Recommendations

This study provides the first region‐specific assessment of total aflatoxin contamination in dairy cattle feed across five major urban centres of Tigray, Ethiopia. Utilising the lateral flow ICT system, the findings reveal that although the average aflatoxin concentration (8.84 µg/kg) was below the national limit of Ethiopia (20 µg/kg), a substantial proportion of samples exceeded both national (22.4%) and EU (29.1%) safety thresholds. The presence of toxigenic fungal species, particularly *A. flavus* and *A. parasiticus*, was the strongest predictor of contamination, alongside key environmental and managerial factors such as storage temperature, prolonged storage duration, high RH, soil‐based flooring and feed composition. Traditional feeds were more heavily contaminated than commercial concentrates, and significant regional differences in contamination levels were observed, with Korem and Alamata exhibiting higher mean concentrations. Alarmingly, the survey revealed low awareness of aflatoxin risks among dairy farmers, limited access to safe storage infrastructure and minimal formal training. These findings collectively demonstrate that aflatoxin contamination in dairy feed is not incidental, but systemic, driven by both biological and socio‐technical vulnerabilities that threaten feed safety, milk quality and public health in the region.

To mitigate aflatoxin risks in Tigray and similar agro‐ecological zones, a coordinated and multi‐level intervention strategy is urgently needed. First, capacity building through targeted farmer training programmmes should be prioritized to improve awareness of aflatoxin sources, prevention methods and the public health implications of contaminated feed and milk. Second, feed storage infrastructure must be upgraded to include moisture‐proof and well‐ventilated storage units with concrete flooring, particularly for smallholder and urban dairy farmers. Third, regulatory agencies and extension services should scale up routine surveillance and enforcement of feed safety standards, including localized testing of both feed and milk for AFB1 and AFM1. Additionally, promoting the use of commercial feed formulations that meet moisture and quality standards may help reduce dependence on high‐risk traditional feedstuffs. From a research perspective, further longitudinal studies are recommended to monitor seasonal trends and the impact of climate variability on aflatoxin prevalence. Finally, fostering cross‐sectoral collaboration between veterinary services, public health authorities, agricultural extension agents and policymakers will be essential for sustaining a safe and productive dairy sector. Without immediate and sustained intervention, aflatoxin contamination will continue to compromise food safety, nutritional security and economic resilience in northern Ethiopia.

## Author Contributions

**Sisay Weldegebriel Zeweld**: conceptualization, methodology, investigation, data curation, formal analysis, writing – original draft, visualization, and project administration. **Meressa Abraha Welearegay**: supervision, resources, writing – review and editing. **Enquebaher Kassaye Tarekegn**: supervision, validation, writing – review and editing.

## Funding

This research was funded by Norwegian Agency for Development Cooperation (NORAD) International Cooperation Program (ICP V) through Mekelle University.

## Ethics Statement

Ethical approval was not required as per Mekelle University Institutional Review Board guidelines for non‐sensitive, low‐risk studies. Informed consent was obtained from all dairy farm owners, who willingly agreed to have their feed tested for total aflatoxin. Sample collection was conducted with their consent, and confidentiality was maintained throughout.

## Conflicts of Interest

The authors declare no conflicts of interest.

## Data Availability

Available from the corresponding author upon reasonable request.

## References

[vms370719-bib-0001] Al‐Jaas, H. , A. Halayqa , and A. Jahajha . 2023. “Aflatoxin Assessment in Food Commodities: Quantification Using ICH‐Validated UPLC‐FLD With Large Flow Cell Volume.” Pharmacy & Pharmacology International Journal 11, no. 5: 186–191.

[vms370719-bib-0002] Álvarez‐Días, F. , B. Torres‐Parga , A. G. Valdivia‐Flores , et al. 2022. “ *Aspergillus Flavus* and Total Aflatoxins Occurrence in Dairy Feed and Aflatoxin M_1_ in Bovine Milk in Aguascalientes, México.” Toxins 14, no. 5: 292. 35622539 10.3390/toxins14050292 PMC9143994

[vms370719-bib-0003] Assaye, M. , N. Gemeda , and G. Weledesemayat . 2016. “ *Aspergillus* Species and Aflatoxin Contamination of Pre and Post‐Harvest Maize Grain in West Gojam, Ethiopia.” Journal of Food Science and Nutrition 2, no. 2: 1–7.

[vms370719-bib-0004] Awuchi, C. G. , E. N. Ondari , C. U. Ogbonna , et al. 2021. “Mycotoxins Affecting Animals, Foods, Humans, and Plants: Types, Occurrence, Toxicities, Action Mechanisms, Prevention, and Detoxification Strategies—A Revisit.” Foods 10, no. 6: 1279. 34205122 10.3390/foods10061279 PMC8228748

[vms370719-bib-0005] Ayelign, A. , and S. De Saeger . 2020. “Mycotoxins in Ethiopia: Current Status, Implications to Food Safety and Mitigation Strategies.” Food Control 113: 107163.

[vms370719-bib-0006] Benkerroum, N. 2020. “Aflatoxins: Producing‐Molds, Structure, Health Issues and Incidence in Southeast Asian and Sub‐Saharan African Countries.” International Journal of Environmental Research and Public Health 17, no. 4: 1215. 32070028 10.3390/ijerph17041215 PMC7068566

[vms370719-bib-0007] Bereka, T. , C. Kuyu , K. Tolera , and E. Addis . 2022. “Current Postharvest Practices and Aflatoxin Contamination Awareness Amongst Maize Producers in Jimma Zone, Southwest of Ethiopia.” World Mycotoxin Journal 15, no. 1: 35–43.

[vms370719-bib-0008] Carvajal‐Campos, A. , A. L. Manizan , S. Tadrist , et al. 2017. “ *Aspergillus Korhogoensis*, a Novel Aflatoxin Producing Species from the Côte d'Ivoire.” Toxins 9, no. 11: 353. 29088078 10.3390/toxins9110353 PMC5705968

[vms370719-bib-0009] Chala, A. , A. Mohammed , A. Ayalew , and H. Skinnes . 2013. “Natural Occurrence of Aflatoxins in Groundnut (*Arachis hypogaea* L.) from Eastern Ethiopia.” Food Control 30, no. 2: 602–605.

[vms370719-bib-0010] Ezekiel, C. , J. Atehnkeng , A. Odebode , and R. Bandyopadhyay . 2014. “Distribution of Aflatoxigenic *Aspergillus* Section *Flavi* in Commercial Poultry Feed in Nigeria.” International Journal of Food Microbiology 189: 18–25. 25108761 10.1016/j.ijfoodmicro.2014.07.026

[vms370719-bib-0011] FAO/WHO . 2004. Safety Evaluation of Certain Mycotoxins in Food. WHO Food Additives Series.

[vms370719-bib-0012] Feddern, V. , G. C. Dors , F. C. de Tavernari , et al. 2013. “Aflatoxins Importance on Animal Nutrition.” In Aflatoxins—Recent Advances and Future Prospects. InTech. 171–195.

[vms370719-bib-0013] Fikadu, J. , B. Tamir , U. Galmessa , and K. Effa . 2022. “Feed Quality, Prevalence of Aflatoxin Contamination in Dairy Feed and Raw Milk in Oromia Special Zone Surrounding Finfinne, Ethiopia.” Asian Journal of Dairy and Food Research 41, no. 1: 8–14.

[vms370719-bib-0014] Fuffa, H. , and K. Urga . 2001. “Survey of Aflatoxin Contamination in Ethiopia.” Ethiopian Journal of Health Sciences 11, no. 1: 17–25.

[vms370719-bib-0015] Geleta, G. S. , A. Nugussa , G. Faye , and G. Ragassa . 2024. “Assessment of Human Health Risks from Aflatoxin M1 in Raw Milk: A Study from North Shewa Zone, Oromia Region, Ethiopia.” Environmental Health Insights 18: 11786302241304524. 39619769 10.1177/11786302241304524 PMC11608451

[vms370719-bib-0016] Gizachew, D. , B. Szonyi , A. Tegegne , J. Hanson , and D. Grace . 2016. “Aflatoxin Contamination of Milk and Dairy Feeds in the Greater Addis Ababa Milk Shed, Ethiopia.” Food Control 59: 773–779.

[vms370719-bib-0017] Guchi, E. 2015. “‘Stakeholders’ Perception about Aflatoxin Contamination in Groundnut (*Arachis hypogaea* L.) Along the Value Chain Actors in Eastern Ethiopia.” International Journal of Food Contamination 2, no. 1: 1–7.

[vms370719-bib-0018] Gurav, N. , and S. Medhe . 2018. “Analysis of Aflatoxins B1, B2, G1 and G2 in Peanuts: Validation Study.” Analytical Chemistry: An Indian Journal 17, no. 2: 126.

[vms370719-bib-0019] Hassan, Z. U. , M. Z. Khan , A. Khan , I. Javed , U. Sadique , and A. Khatoon . 2012. “Ochratoxicosis in White Leghorn Breeder Hens: Production and Breeding Performance.” Pakistan Veterinary Journal 32, no. 4: 557–561.

[vms370719-bib-0020] Kemboi, D. C. , G. Antonissen , P. E. Ochieng , et al. 2020. “A Review of the Impact of Mycotoxins on Dairy Cattle Health: Challenges for Food Safety and Dairy Production in Sub‐Saharan Africa.” Toxins 12, no. 4: 222. 32252249 10.3390/toxins12040222 PMC7232242

[vms370719-bib-0021] Kortei, N. K. , R. A. Tetteh , M. Wiafe‐Kwagyan , D. N. K. Amon , and G. T. Odamtten . 2022. “Mycobiota Profile, Phenology, and Potential Toxicogenic and Pathogenic Species Associated With Stored Groundnuts (*Arachis hypogaea* L.) from the Volta Region, Ghana.” Food Science & Nutrition 10, no. 3: 888–902. 35311164 10.1002/fsn3.2719 PMC8907750

[vms370719-bib-0022] Kraus, S. , B. Cvak , and L. Fidalgo . 2024. “Validation of the AgraStrip Pro Total Aflatoxin WATEX Method for Detection of Total Aflatoxins in Corn and Peanut Paste: AOAC Performance Tested Method SM 032402.” Journal of AOAC International 107, no. 4: 641–648. 38741217 10.1093/jaoacint/qsae040

[vms370719-bib-0023] Krska, R. , J. L. Richard , R. Schuhmacher , A. B. Slate , and T. B. Whitaker . 2012. Romer Labs Guide to Mycotoxins. 4th ed. Romer Labs Diagnostic GmbH. 1–56.

[vms370719-bib-0024] Mahato, D. K. , K. E. Lee , M. Kamle , et al. 2019. “Aflatoxins in Food and Feed: An Overview on Prevalence, Detection and Control Strategies.” Frontiers in Microbiology 10: 2266. 31636616 10.3389/fmicb.2019.02266 PMC6787635

[vms370719-bib-0025] Medina, Á. , J. M. González‐Jartín , and M. J. Sainz . 2017. “Impact of Global Warming on Mycotoxins.” Current Opinion in Food Science 18: 76–81.

[vms370719-bib-0026] Mengesha, G. , T. Bekele , H. Ashagrie , and A. Z. Woldegiorgis . 2024. “Level of Aflatoxins in Dairy Feeds, Poultry Feeds, and Feed Ingredients Produced by Feed Factories in Addis Ababa, Ethiopia.” Mycotoxin Research 40, no. 2: 309–318. 38530632 10.1007/s12550-024-00531-8

[vms370719-bib-0027] Mohammed, A. , A. Chala , M. Dejene , et al. 2016. “Aspergillus and Aflatoxin in Groundnut (*Arachis hypogaea* L.) and Groundnut Cake in Eastern Ethiopia.” Food Additives & Contaminants: Part B 9, no. 4: 290–298. 10.1080/19393210.2016.1216468 27748169

[vms370719-bib-0028] Mohammed, A. , P. C. Faustinelli , A. Chala , et al. 2021. “Genetic Fingerprinting and Aflatoxin Production of Aspergillus Section Flavi Associated With Groundnut in Eastern Ethiopia.” BMC Microbiology 21, no. 1: 239. 34454439 10.1186/s12866-021-02290-3 PMC8403416

[vms370719-bib-0029] Motbaynor Wubaye, A. , D. D. Kassaye , and D. M. Keffale . 2021. “Magnitude of Aflatoxigenic *Aspergillus* Species, Level of Aflatoxin B1 and Associated Factors in Stored Feed at Poultry Farms in Dire Dawa, Ethiopia.” Veterinary Medicine International 2021: 6638083. 10.1155/2021/6638083. 34721834 PMC8553476

[vms370719-bib-0030] Nishimwe, K. , J. A. L. Mandap , and G. P. Munkvold . 2020. “Advances in Understanding Fungal Contamination in Cereals.” In Advances in Postharvest Management of Cereals and Grains. Burleigh Dodds Science Publishing. 31–66.

[vms370719-bib-0031] Sánchez‐Bayo, F. , L. de Almeida , R. Williams , G. Wright , I. R. Kennedy , and A. Crossan . 2017. “The Aflatoxin Quicktest, a Practical Tool for Ensuring Safety in Agricultural Produce.” In Poisoning, from Specific Toxic Agents to Novel Rapid and Simplified Techniques for Analysis. IntechOpen. 10.5772/intechopen.71331.

[vms370719-bib-0032] Shad, Z. M. , M. Ghavami , and G. G. Atungulu . 2019. “Occurrence of Aflatoxin in Dairy Cow Feed Ingredients and Total Mixed Ration.” Applied Engineering in Agriculture 35, no. 5: 679–686.

[vms370719-bib-0033] Tadele, F. , B. Demissie , A. Amsalu , et al. 2023. “Aflatoxin Contamination of Animal Feeds and its Predictors among Dairy Farms in Northwest Ethiopia: One Health Approach Implications.” Frontiers in Veterinary Science 10: 1123573. 37035821 10.3389/fvets.2023.1123573 PMC10076730

[vms370719-bib-0034] Tefera, W. , G. E. Vegarud , M. Taye , and T. Taye . 2022. “Aflatoxin Contamination in Dairy Feed During Wet and Dry Seasons in Selected Rural Areas of Sidama Zone in Southern Ethiopia.” International Journal of Agricultural Science and Food Technology 8, no. 1: 078–082.

[vms370719-bib-0035] Tesfaye, A. , M. Y. Kurtu , Y. Y. Mummed , and A. Mohammed . 2024. “Aflatoxins Levels in Concentrate Feeds Collected From Specialized Dairy Farms and Local Markets in Selected Urban Centers of Eastern Ethiopia.” Toxins 16, no. 10: 418. 39453194 10.3390/toxins16100418 PMC11511069

[vms370719-bib-0036] Thrusfield, M. 2018. Veterinary Epidemiology. John Wiley & Sons.

[vms370719-bib-0037] Toma, A. 2019. “Knowledge, Attitude and Practice of Farmers towards Aflatoxin in Cereal Crops in Wolaita Zone, Southern Ethiopia.” EC Nutrition 14: 247–254.

[vms370719-bib-0038] Wu, F. 2015. “Global Impacts of Aflatoxin in Maize: Trade and Human Health.” World Mycotoxin Journal 8, no. 2: 137–142.

[vms370719-bib-0039] Yakubu, A. , and A. Vyas . 2020. “Aflatoxin: Occurrence, Regulation, and Detection in Food and Feed.” In Microbial Biotechnology: Basic Research and Applications. Springer Nature. 337–353.

[vms370719-bib-0040] Yegrem, L. 2020. “Aflatoxins Contaminations Levels in Animal Feeds and Milk around Addis Ababa, Ethiopia.” Academic Research Journal of Agricultural Science and Research 8, no. 3: 174–188.

[vms370719-bib-0041] Zeweld, S. W. , E. K. Tarekegn , and M. A. Welearegay . 2025. “Detection of Aflatoxigenic Fungi in Dairy Cattle Feed: Findings from Urban Districts of Tigray, Ethiopia.” Veterinary Medicine and Science 11, no. 3: e70362. 10.1002/vms3.70362. 40323976 PMC12051845

